# Book Notices

**Published:** 1893-03

**Authors:** 


### Book Notices.

NOTES ON THE NEWER REMEDIES, their therapeutic applications and modes of administration, by David Cerna, M. D., Ph. D., Demonstrator of Physiology, Medical Department, University of Texas; formerly Assistant in Physiology, Demonstrator of and Lecturer on Experimental Therapeutics, University of Pennsylvania. W. B. Saunders, Philadelphia, 1892.

In this excellently well-gotten-np little brochure, a veritable "*multum in parvo*," Dr. Cerna's aim "has been to furnish the practitioner and student, in as brief a manner as possible, the most salient points concerning the employment of the newer drugs in the treatment of disease." That the author's purpose has been accomplished in a most satisfactory manner, is attested not only by the hearty reception and richly deserved laudation accorded his work at home and abroad, but by the further fact that, in one hundred and sixty-seven octavo pages, he has given, in alphabetical order, the origin, chemical formulæ, physical properties, solubility, therapeutic application, and modes of administration, paying special attention to the two latter subjects, of over three hundred of the most important of the "newer remedies," "whose usefulness has been more or less ascertained by clinical investigation," thus constituting the most "newsy" and valuable ready-reference hand-book on the subject extant, one that should be in the hands of every physician wishing to give his patients the benefit of recent discoveries in therapeutics. The present volume, replete with information to be obtained from no other single book, copiously indexed, using both the apothecaries and the mitric systems of weights. filling, as it certainly does, to completeness, "a long felt want," will not only be revised, as the author assures us, as often as the progress of scientific therapeutics shall demand it, but is destined, also, as the basis of a larger, a more comprehensive treatise, in which the physiological and therapeutical actions of the new medicaments will be fully discussed;—a work for which Dr. Cerna is pre-eminently qualified. R. H. L. BIBB, M. D.

---

A TEXAS BOOK: "FERMENTATION, INFECTION AND IMMUNITY. —A new theory of these processes, which unifies their primary causation and places the explanation of their phenomena in Chemistry, Biology and the Dynamics of Molecular Physics. By J. W. McLaughlin, M. D., Austin, Texas. Cloth: price $2.50. Printed in Austin, by E. Von Boeckmann, Steam Power Press.

This is a highly scientific work; too deep and abstruse for the general reader, and one which we confess our inability to criticise or review, as it should be done. The style is clear and forcible, and the conclusions, arrived at by logical deductions, appear to us incontrovertible. The work ran through the *Texas Sanitarian* as a serial, and notwithstanding the comparatively wide circulation of that publication,—27,000 copies in the year, —it has received but little attention. Had it been written by some foreigner, with an unpronouncable name, no doubt certain toady Northern journals, which see so much to admire in everything foreign, and so little in anything Southern in its origin, the author would have received much commendation.

Dr. McLaughlin has studied the subject of *immunity* very closely; and being not satisfied with either of the existing theories by which it is sought to be explained, evolved one of his own, which he calls the "physical theory," and accounts for immunity in a highly scientific manner, through the doctrine of what he calls "interference." We have read his book carefully, and as stated, we are unable to closely criticise it; we think his theory more rational, and his position more tenable than any other proposed.

---

A TREATISE ON DISEASES OF THE RECTUM, ANUS AND SIGMOID FLEXURE. By Joseph Matthews, M. D., etc., etc. Published by D. Appleton & Co., New York. Price, —

The author says that he wrote this book because he desired to record his individual experience as a rectal specialist for the past fifteen years, and in answer to the demand of his students and friends, and he has done his work well in every respect, and has made a book that covers the entire ground that he has laid out. It is a book that will interest the general practitioner, as well as the specialist, in fact, the general practitioner who studies it closely and follows its instruction, need not fear to undertake the treatment of any and all the diseases and conditions treated of. It is a book that ought to be in the library of every practitioner, who desires to keep abreast of the times. The work is stamped with the individuality of the writer, and is wholly his own. He has introduced several chapters new to books on this subject. He has devised and practiced a new operation for fistula in ano, which certainly is practical, and must prove the coming operation for this trouble. The chapter on the anatomy of the rectum in relation to the reflexes is made to follow that of the hysterical rectum, in order to account for some vague affections of the lower bowel. And by the way, the subject of the reflexes is one of vast importance, perhaps of more importance and of more interest than any other subject that we have to deal with. Perhaps the field of gynecology reveals more evidence of reflex action than any other. Yet those engaged in other specialties have found a great deal to interest them from the subject under discussion, and it is charged that the specialist is likely to refer the patient's trouble to the part of the anatomy that he treats.

The publishers have gotten the book up in good shape. The type is large and clear, and the paper smooth, heavy, and fine, and it is bound in first-class shape. W. E. S.

---

BARTHOLOW'S HYPODERMATIC MEDICATION. The fact that this book has gone through five editions in so short a time, shows that the work is deservedly, popular. The book throughout is well written. The mechanical part of the work is done first-class, type large, clear-cut and plain, so that he who runs (and who does not in this age of push) may read. The paper is good, and the binding well done; in fact, it is first-class in every respect. The author, in the first chapter, gives a history of subcutaneous medication. Next, the method of use in, and the syringe, and how to care for it, etc., and the probable dangers from its use. This work is a full and complete history of all that is known in regard to this method of administering medicine. This edition brings the system up to '91. W. E. S.

---

BRYCE'S POCKET PRACTICE, a complete and condensed work on the Practice of Medicine, for Physicians and Students. By Clarence A. Bryce, M. D., editor Southern Clinic, etc. Richmond, Va. Price, $1.

This book, which is intended as a pocket companion, is intended to "refresh the memory" on many points. It contains, in a concise form, a synopsis of the usual contents of standard works on practice,—symptomatology, diagnosis, etc.,—and an outline of treatment, full enough for practical purposes, the treatment being from the standpoint of Dr. Bryce's own observation and experience,—*his* method of curing sick people. The book will be found useful, more particularly to one busily engaged in practice.

---

TEXT-BOOK OF OPHTHALMOLOGY. By Dr. Ernest Fuchs, Professor of Ophthalmology in the University of Vienna. Authorized translation from the second enlarged and improved German edition. By A. Duane, M. D., Assistant Surgeon

Ophthalmia and Aural Institute, New York. With numerous illustrations. New York: D. Appleton & Co. 1892.

It seems there is no end "of the making of many books." The one before us is handsomely gotten up, and handsomely illustrated. In its text, so far as we have been able to examine it, we see no radical difference between this and several other works on the subject. This one appears to have been written "from the standpoint of experience," and every oculist's experience must be pretty much alike in the main. It is a nice book to have, provided your library is not supplied with any other on the same subject, but we cannot say that it is any better than those we have already noticed, "which have gone before." The publishers always do their part well.

---

Blakiston's Sons announce that very soon they will issue a revised edition of Moultin's Text-Book of Surgery, edited by Dr. John B. Hamilton. It will present *Surgery as it is to-day*, with new and beautifully colored illustrations printed in with the text. The publishers claim that this is the best text-book for the student and for general reference by practitioners. More than twenty medical colleges recommended it for a text-book, and the first edition, a very large one, was rapidly exhausted.

---

LUNGS, HEART AND KIDNEYS. Ready Reference Series. N. S. Davis, Jr. The F. A. Davis Pub. Co.

This book is one which the writer has condensed and given only that which is practical and necessary to the understanding of the subject under consideration. He has paid especial attention to the treatment of the diseases of these organs, and left out matters that are in dispute or doubtful. It is practical and common-sense. The publishers have, as usual, done their work well. The binding is good, paper not heavy but fine, and the type clear-cut and plain as type can be. W. E. S.

---

DISEASES OF THE KIDNEYS AND BLADDER. McNutt. Lippincott & Co.; Phila., Pa.

This book is based upon the notes of lectures delivered to the medical students of the University of California, and is what is demanded by student and practitioner, in this age of advancement; that is, it is full enough to give a clear idea of the anatomy and physiology of the organs of which it treats, without which it would hardly come up to the demands of the profession, for in order to treat diseases of these organs intelligently, it is necessary to understand their anatomy and physiology. It brings the treatment of these organs down to the latter part of '92, or the very latest. The publishers have done their work well. The book is cheap at $2.50, the price asked for it.

W. E. S.

---

MANUAL OF PRACTICAL MEDICAL AND PHYSIOLOGICAL CHEMISTRY. Charles E. Pellew. 1892. D. Appleton & Co., Publiahers.

This book is just what the title says it is, and is what every student of medicine needs, and many M. D.'s would be greatly benefited by studying it carefully. The student will get as much, if not more, pure physiological chemistry, with foodstuffs and their products of assimilation, and with the different fluids and tissues of the body, but that, whenever possible, particular attention has been paid to the latest clinical tests. Buy it; read it. It will do you good, as well as your patients.

